# Comparison of inflammatory indices in patients with metastatic non-small cell lung cancer

**DOI:** 10.1590/1806-9282.20240411

**Published:** 2025-06-02

**Authors:** Zuhat Urakçı, Zeynep Oruc, Halil Kömek, Senar Ebinç, Esra Pirinççi, Ziya Kalkan, Bekir Taşdemir, Muhammet Ali Kaplan, Mehmet Küçüköner, Abdurrahman Işıkdoğan

**Affiliations:** 1 Dicle University, Faculty of Medicine, Department of Medical Oncology – Diyarbakır, Türkiye.; 2 Gazi Yasargil Training and Research Hospital, Department of Nuclear Medicine – Diyarbakır, Türkiye.; 3 Dicle University, Faculty of Medicine, Department of Internal Medicine – Diyarbakır, Türkiye.; 4 Dicle University, Faculty of Medicine, Department of Nuclear Medicine – Diyarbakır, Türkiye.

**Keywords:** Non-small cell lung cancer, Inflammation, Inflammatory markers, Prognosis, Correlation

## Abstract

**OBJECTIVE:**

The aim of this study was to compare the prognostic value of inflammatory indices, including the prognostic nutritional index, advanced lung cancer inflammation index, neutrophil/lymphocyte ratio, platelet/lymphocyte ratio, Glasgow Prognostic Score, and C-reactive protein/albumin ratio, in patients with metastatic non-small cell lung cancer and to determine whether any of the indices are correlated.

**METHODS:**

The demographic, clinicopathological, and laboratory data of 179 patients with metastatic non-small cell lung cancer who presented to our medical oncology clinic between 2014 and 2021 were retrospectively obtained from the hospital database system.

**RESULTS:**

Among the inflammatory indices, C-reactive protein/albumin ratio (p=0.002), advanced lung cancer inflammation index (p=0.04), and neutrophil/lymphocyte ratio (p=0.008) were associated with overall survival. Multivariate overall survival analysis showed that Eastern Cooperative Oncology Group Performance Status score (HR 1.37; 95%CI 1.06–1.77; p=0.016) and C-reactive protein/albumin ratio (HR 1.13; 95%CI 1.03–1.24; p=0.01) were independent prognostic factors associated with overall survival. Patients with a high and low C-reactive protein/albumin ratio differed statistically significantly in terms of gender, histopathological subtype, history of smoking, and metastatic site (p<0.05). Correlation analysis showed that C-reactive protein/albumin ratio was negatively correlated with advanced lung cancer inflammation index (r=-0.433, p<0.001) and positively correlated with the other four indices (neutrophil/lymphocyte ratio: r=0.368, p<0.001; platelet/lymphocyte ratio: r=0.321, p<0.001; Glasgow Prognostic Score: r=0.537, p<0.001; and prognostic nutritional index: r=0.223, p=0.003).

**CONCLUSION:**

Our findings show that among the investigated inflammatory indices, C-reactive protein/albumin ratio has the highest prognostic value in patients with metastatic non-small cell lung cancer.

## INTRODUCTION

Lung cancer is included among the cancers with a high mortality rate. Despite significant advances in the treatment of lung cancer, its prognosis remains poor^1,2^. Although many prognostic factors (age, gender, performance status, histopathological type, history of smoking, disease stage, number of metastatic sites, treatment history, and molecular markers) have been identified in patients with metastatic non-small cell lung cancer (NSCLC), there is a prevailing need for new prognostic factors with greater value for determining the course of disease, optimizing the choice of treatment, and avoiding unnecessary treatments.

Recently, numerous studies have investigated the ability of nutrition and inflammatory indices to predict the prognosis in patients diagnosed with metastatic lung cancer, including the prognostic nutritional index (PNI)^3^, advanced lung cancer inflammation index (ALI)^4^, neutrophil/lymphocyte ratio (NLR)^5^, platelet/lymphocyte ratio (PLR)^6^, Glasgow Prognostic Score (GPS)^7^, and C-reactive protein/albumin ratio (CAR)^8^.

Numerous inflammation-based prognostic scores have been evaluated alone and in combination in patients with various cancers^9–11^; however, they have been generally inconsistent^12,13^. The present study aimed to compare the prognostic value of six inflammatory indices and to determine their correlation in patients diagnosed with metastatic NSCLC.

## METHODS

### Patient population

The study included 179 patients diagnosed with metastatic NSCLC who presented to the Dicle University Medical Oncology Clinic, Diyarbakır, Turkey, between January 2015 and February 2021, and had complete medical records. Patient demographic characteristics, clinicopathological and laboratory findings, including the white blood cell (WBC) count, total lymphocyte count, total neutrophil count, platelet count, hemoglobin, serum albumin, and C-reactive protein (CRP) levels, and data pertaining to their treatment were retrospectively obtained from the hospital record system and were analyzed.

Patients with histopathologically confirmed NSCLC who were accepted as Stage IV based on TNM-8 classification and met the study's inclusion criteria were included in the study. Patients with acute or chronic inflammatory diseases (acute/chronic infection and collagen vascular diseases, such as rheumatoid arthritis and systemic lupus erythematosus), liver disease, a history of steroid therapy, and a history of immunosuppressive therapy were excluded from the study.

### Systemic inflammatory markers

Complete blood count parameters (neutrophil, lymphocyte, monocyte, platelet, and hemoglobin) and biochemical parameters (CRP and albumin) were obtained via routine blood testing. Only blood tests performed during the 28 days preceding the initial administration of cancer treatment were taken into consideration. The inflammatory indices used in this study (NLR, PLR, ALI, GPS, PNI, and C-reactive protein/albumin [CAR]) were computed based on published formulae, as follows: PNI: 10 ’ albumin (g dL^-1^)+0.005 ’ lymphocyte count (lymphocytes, mm^-3^); NLR: neutrophil count (neutrophils, mm^-3^)/lymphocyte count; PLR: platelet count (platelets, mm^-3^)/lymphocyte count; ALI: body mass index′ albumin (g dL^-1^)/NLR; GPS: CRP ≤10 mg l^-1^ (0), CRP >10 mg l^-1^ (1), and CRP >10 mg l^-1^ and albumin <35 g l^-1^ (2).

### Statistical analysis

Overall survival (OS) is defined as the length of time from the initial diagnosis of a disease to the last follow-up or death due to any cause. Statistical analysis was performed using IBM SPSS Statistics for Windows v.25.0 (IBM Corp. Armonk, NY, USA). The Mann-Whitney U test was used to compare two independent groups of quantitative data. Spearman's rho was used for the analysis of correlations between the variables. Pearson's chi-square test and/or Fisher's exact test were used to compare categorical variables. The effect of OS parameters was analyzed using either the Kaplan-Meier test or the Mantel-Cox log-rank test. Cox regression analysis was performed using significant independent variables in order to estimate the effects of prognostic variables on survival. Variables were analyzed at the 95%CI, and the level of statistical significance was set at p<0.05.

## RESULTS

### Patient characteristics

Among the 179 patients, 151 (84.4%) were male and 28 (15.6%) were female. All patients had metastatic NSCLC. Detailed demographic and clinicopathological characteristics are summarized in Table 1. The median OS was 10 months (95%CI 2–88). Univariate analysis of the effects of clinicopathological characteristics, laboratory parameters, and inflammatory indices on OS showed that Eastern Cooperative Oncology Group Performance Status (ECOG PS) score (p=0.004), the presence of driver mutations (p=0.011), and the inflammatory indices (CAR: p=0.002; ALI: p=0.04; and NLR: p=0.008) were associated with OS. Multivariate OS analysis showed that an ECOG PS score ≥2 (hazard ratio [HR] 1.37; 95%CI 1.06–1.77; p=0.016) and CAR >1 (HR 1.13; 95%CI 1.03–1.24; p=0.01) were independent prognostic factors for OS (Table 2). The ECOG PS score from among the clinical prognostic factors and CAR from among the inflammatory markers were found to be independent predictors of OS.

**Table 1:** Patients’ clinicopathological characteristics.

		n=179 (%)
Age (years) (median, range)		64 (27–84)
Gender		
	Female	28 (15.6)
	Male	151 (84.4)
ECOG performance status		
	0–1	127 (70.9)
	≥2	52 (29.1)
Tumor localization		
	Right	101 (56.4)
	Left	78 (43.6)
Histopathological subtype		
	Adenocarcinoma	130 (72.6)
	Squamous cell carcinoma	38 (21.3)
	Other	11 (6.1)
Metastatic sites		
	Liver	6 (3.4)
	Brain	8 (4.5)
	Adrenal	9 (5)
	Lung	21 (11.7)
	Bone	26 (14.5)
	Multiple	86 (48)
	Other sites	23 (12.9)
EGFR mutation positivity		26 (14.5)
ALK mutation positivity		4 (2.2)
First-line treatment (n=150)		
	Chemotherapy	127 (84.4)
	Erlotinib	21 (11.7)
	Alectinib	2 (1.1)
Smoking		
	Yes	119 (66.5)
	No	32 (17.9)
	Unknown	28 (15.6)
Comorbid disease (n=53)		
	Diabetes mellitus	14 (7.8)
	Hypertension	23 (12.8)
	Coronary artery disease	16 (8.9)
Hemoglobin (g/dL)		
	≤11	22 (12.3)
	>11	157 (87.7)
CAR		
	≤1	88 (49.2)
	>1	91 (50.8)
ALI		
	≤21.4	97 (54.2)
	>21.4	82 (45.8)
NLR		
	≤3.5	88 (49.2)
	>3.5	91 (50.8)

NLR: neutrophil/lymphocyte ratio; ALI: advanced lung cancer inflammation index; CAR: C-reactive protein/albumin ratio; ECOG: Eastern Cooperative Oncology Group; EGFR: epidermal growth factor receptor; ALK: anaplastic lymphoma kinase.

**Table 2:** Univariate and multivariate analyses of prognostic factors for overall survival.

		Univariate analysis			Multivariate analysis		
		HR	95%CI	p-value	HR	95%CI	p-value
Age at diagnosis		1.01	0.99–1.02	0.155			
Gender (female/male)		0.68	0.44–1.05	0.088			
ECOG PS (0–1/≥2)		1.45	1.12–1.87	**0.004**	1.37	1.06–1.77	**0.016**
Tumor localization (right/left)		1.06	0.77–1.47	0.69			
Presence of driver mutations		0.48	0.27–0.84	**0.011**			
Histopathological subtype		0.99	0.81–1.22	0.99			
Metastatic site		0.01	0.91–1.11	0.84			
Smoking		1.40	0.92–2.14	0.115			
Hemoglobin level		0.96	0.88–1.05	0.40			
GPS		1.18	0.94–1.47	0.14			
PLR		1.00	0.99–1.01	0.98			
PNI		1.00	0.99–1.02	0.21			
CAR		1.15	1.05–1.26	**0.002**	1.13	1.03–1.24	**0.010**
	Low (≤1) (n=88)						
	High (>1) (n=91)						
ALI		0.98	0.97–1.00	**0.04**			
	Low (≤21.4) (n=97)						
	High (>21.4) (n=82)						
NLR		1.07	1.01–1.12	**0.008**			
	Low (≤3.5) (n=88)						
	High (>3.5) (n=91)						

GPS: Glasgow Prognostic Score; PLR: platelet-to-lymphocyte ratio; PNI: Prognostic nutritional index; NLR: neutrophil/lymphocyte ratio; ALI: Advanced lung cancer inflammation index; CAR: C-reactive protein/albumin ratio; HR: hazard ratio; CI: confidence interval; ECOG PS: Eastern Cooperative Oncology Group Performance Status. Statistically significant values are denoted in bold.

Receiver operating characteristic (ROC) curve analysis of the inflammatory indices associated with OS showed the following: the cutoff value for CAR was ≤1/<1, with a sensitivity of 58% and a specificity of 57% (area under the curve [AUC]: 0.633 [0.552–0.715], p=0.002); the cutoff value for ALI was ≤21.4/<21.4, with a sensitivity of 43% and a specificity of 42% (AUC: 0.377 [0.295–0.460], p=0.005); the cutoff value for NLR was ≤3.5/<3.5, with a sensitivity of 62% and a specificity of 62% (AUC: 0.61 [0.549–0.713], p=0.003). When the patients were categorized into two groups based on the cutoff values of these three indices, the median OS was 13 months in patients with a CAR ≤1 and 9 months in those with a CAR >1 (p=0.009), the median OS was 9 months in patients with an ALI ≤21.4 and 13 months in those with an ALI >21.4 (p=0.018), and the median OS was 8 months in patients with an NLR ≤3.5 and 13 months in those with an NLR >3.5 (p=0.003).

### Analysis of C-reactive protein/albumin ratio

Based on the ROC curve analysis, 91 (50.8%) patients had a high CAR (>1) and 88 (49.1%) had a low CAR (≤1) (CAR cutoff value: 1). The distribution of the clinicopathological characteristics of the patients with a low and high CAR is presented in Table 3. These two groups of patients differed significantly in terms of gender, histopathological subtype, history of smoking, and metastatic site. Patients who were male, those who had squamous cell carcinoma, those with a positive history of smoking, and those with multiple metastases had a higher CAR (p<0.05).

**Table 3:** Distribution of clinicopathological characteristics across C-reactive protein/albumin ratio groups.

		All patients n (%)	CAR		p-value
			Low, n=88 (49.1%)	High, n=91 (50.8%)	
Age (mean±std. dev.)		63.5±11.6	63.9±12.2	63.2±11.2	0.69*
Gender					
	Female	28 (15.6)	20 (71.4)	8 (28.6)	**0.01** **
	Male	151 (84.4)	68 (45)	83 (55)	
ECOG performance status					
	0–1	127 (70.9)	63 (52.1)	58 (47.9)	0.15**
	≥2	52 (29.1)	21 (40.4)	31 (59.6)	
Tumor localization					
	Right lung	101 (56.4)	49 (48.5)	52 (51.5)	0.93**
	Left lung	78 (43.6)	34 (47.9)	37 (52.1)	
Histopathological subtype					
	Adenocarcinoma	130 (72.6)	69 (53.1)	61 (46.9)	**0.04** **
	Squamous cell carcinoma	38 (21.3)	12 (31.6)	26 (68.4)	
	Other	11 (6.1)	7 (63.6)	4 (36.4)	
EGFR mutation (n=78)					
	Yes	26 (33.3)	18 (69.2)	8 (30.8)	0.19**
	No	52 (66.7)	28 (53.8)	24 (46.2)	
ALK mutation (n=29)					
	Yes	4 (13.8)	1 (25)	3 (75)	0.29***
	No	25 (86.2)	15 (60)	10 (40)	
First-line treatment (n=150)					
	Chemotherapy	127 (84.4)	61 (48)	66 (52)	0.12**
	Targeted therapy	23 (17.7)	15 (65.2)	8 (34.8)	
Smoking history					
	Yes	119 (66.5)	50 (42)	69 (58)	**0.01** **
	No	32 (17.9)	23 (71.9)	9 (28.1)	
	Unknown	28 (15.6)	15 (53.6)	13 (46.4)	
Comorbid disease (n=53)					
	Diabetes mellitus (yes)	14	6 (42.9)	8 (57.1)	0.66**
	Hypertension (yes)	23	12 (52.2)	11 (47.8)	0.71**
	Coronary artery disease (yes)	16	7 (43.8)	9 (56.3)	0.88**
Metastatic site					
	Visceral	44 (24.6)	30 (68.2)	14 (31.8)	**0.005** *
	Non-visceral	49 (27.4)	25 (51)	24 (49)	
	Multiple	86 (48)	33 (38.4)	53 (61.6)	

* t-test,

** chi-square test,

*** Fisher's exact test. CAR; C-reactive protein/albumin ratio;

ECOG: Eastern Cooperative Oncology Group. Statistically significant values are denoted in bold.

### Correlation analysis

Spearman's correlation analysis showed that the majority of the inflammatory indices were positively or negatively correlated with each other. Among the inflammatory indices, ALI had a statistically significant negative correlation with the other five indices (p<0.001). Additionally, CAR had a statistically significant negative correlation with ALI (r=-0.433, p<0.001) and statistically significant positive correlations with the other four indices (NLR: r=0.368, p≤0.001; PLR: r=0.321, p<0.001; GPS: r=0.537, p<0.001; and PNI: r=0.223, p=0.003).

## DISCUSSION

The present study compared the prognostic value of six inflammatory indices (NLR, PLR, CAR, ALI, PNI, and GPS) in patients diagnosed with metastatic NSCLC and investigated whether any of them correlated with each other. As previously reported, the present study findings confirm the prognostic role of ECOG PS and the presence of driver mutations in patients with metastatic NSCLC. Moreover, among the six inflammatory indices examined, only CAR (p=0.002), ALI (p=0.04), and NLR (p=0.008) were observed to be associated with OS. Multivariate OS analysis showed that CAR had the greatest prognostic value, as compared to the other five inflammatory markers (HR 1.13; 95%CI 1.03–1.24; p=0.01). Our findings also confirm that survival time in metastatic NSCLC patients is linked to systemic inflammation. Furthermore, a low CAR (≤1) was associated with longer OS (p=0.009).

The literature includes only a limited number of studies that compare the prognostic value of inflammatory markers and evaluate their relationships with each other in NSCLC patients. An earlier study that assessed the prognostic significance of NLR, PLR, ALI, and the lymphocyte-to-monocyte ratio (LMR) in NSCLC patients reported that a high NLR, a high PLR, a low LMR, and a low ALI were associated with a poor OS^14^; however, the researchers did not compare the prognostic value of the inflammatory indices.

Similar to the present study, a study on Chinese metastatic NSCLC patients compared multiple inflammatory indices (GPS, Modified Glasgow Prognostic Score [mGPS], NLR, PLR, LMR, and CAR) and observed that NLR (HR 1.52; 95%CI 1.18–1.96; p=0.001) and CAR (HR 1.82; 95%CI 1.45–2.2; p<0.001) were better prognostic factors than the other indices. In their study, the cutoff value for CAR was 0.235. It also reported that males and patients with squamous cell carcinoma had higher CAR values (p<0.05), as in the present study. In addition, the researchers reported that CAR was positively correlated with all the other inflammatory indices studied (GPS, mGPS, NLR, PLR, and LMR)^8^. The cutoff value for the optimal CAR remains unknown. CAR cutoff values reported in earlier studies are inconsistent. In the present study, the CAR cutoff value was determined to be 1 via ROC curve analysis. Inconsistent and arbitrarily defined cutoff values of various inflammatory markers in the literature can lead to incorrect assessments and complicate the use of these parameters in routine clinical practice.

The present study also evaluated the relationship between CAR and clinicopathological characteristics. Male gender, squamous histology, a positive history of smoking, and the presence of multiple metastases were associated with a high CAR (p<0.05). Smoking exacerbates chronic inflammation and increases the CRP level. Generally, habitual smoking is more common among males than females. Furthermore, squamous cell carcinoma is closely associated with regular smoking^15^. A positive history of smoking might explain the present study's observation of a correlation between CAR and clinicopathological characteristics. In addition, CAR was also higher in our patients who had multiple metastases. A high CRP level in metastatic NSCLC patients is indicative of tissue destruction and inflammation associated with metastasis, as well as the extent of invasion^16^.

The literature includes very limited data pertaining to the correlations between inflammatory indices. Relevant studies have failed to elucidate the value of inflammatory indices for the prediction of the prognosis in NSCLC patients, as well as to determine which index or indices should be used for predicting the prognosis due to the inclusion of heterogeneous patient populations, study limitations, confounding results, and inconsistent findings. Our findings show that there is a correlation between the studied inflammatory indices. Spearman's rho correlation analysis showed that CAR, in particular, was negatively correlated with ALI and positively correlated with the other studied inflammatory indices, with statistical significance, as follows: ALI (r=-0.433, p<0.001); NLR (r=0.368, p<0.001); PLR (r=0.321, p<0.001); GPS (r=0.537, p<0.001); and PNI (r=0.223, p=0.003). The strong correlation between inflammatory indices based on cellular and biochemical parameters shows that there is a complex interaction between the host immune system, immune system response, and the inflamed tumor microenvironment^17^.

The present study has some limitations, including a single-center retrospective design and a small patient population. In addition, even though the present study shows that CAR is an independent predictor of prognosis in metastatic NSCLC patients, the specificity and sensitivity of CAR were not high. As such, additional large-scale multicentric studies are needed to confirm the present findings. Other limitations are the inclusion of a heterogeneous patient group, variability in clinical outcomes resulting from the heterogeneity of patient treatments, and the combined evaluation of patients with and without driver mutations.

In conclusion, our findings show that CAR is an independent prognostic factor in metastatic NSCLC patients and is a stronger prognostic factor than other inflammatory indices studied. If this finding is confirmed by larger prospective studies, CAR could be used to help clinicians predict the prognosis in metastatic NSCLC patients, as it is an index that is easy to use and is widely available in clinical practice.
